# Gene Prioritization of Resistant Rice Gene against *Xanthomas oryzae pv. oryzae* by Using Text Mining Technologies

**DOI:** 10.1155/2013/853043

**Published:** 2013-11-25

**Authors:** Jingbo Xia, Xing Zhang, Daojun Yuan, Lingling Chen, Jonathan Webster, Alex Chengyu Fang

**Affiliations:** ^1^College of Science, Huazhong Agricultural University, Wuhan 430070, Hubei, China; ^2^Department of Chinese, Translation and Linguistics, City University of Hong Kong, Kowloon, Hong Kong; ^3^The Halliday Centre for Intelligent Applications of Language Studies, City University of Hong Kong, Kowloon, Hong Kong; ^4^College of Plant Science and Technology, Huazhong Agricultural University, Wuhan 430070, Hubei, China; ^5^College of Life Science, Huazhong Agricultural University, Wuhan 430070, Hubei, China

## Abstract

To effectively assess the possibility of the unknown rice protein resistant to *Xanthomonas oryzae pv. oryzae*, a hybrid strategy is proposed to enhance gene prioritization by combining text mining technologies with a sequence-based approach. The text mining technique of term frequency inverse document frequency is used to measure the importance of distinguished terms which reflect biomedical activity in rice before candidate genes are screened and vital terms are produced. Afterwards, a built-in classifier under the chaos games representation algorithm is used to sieve the best possible candidate gene. Our experiment results show that the combination of these two methods achieves enhanced gene prioritization.

## 1. Introduction

Due to the availability of abundant genomic resources, rice has become a model species for the genomic study. Taking into account that rice has been the main food for a large section of the world population, research issues related to yielding and antidisease have drawn much attention [1]. Bacterial blight, caused by *Xanthomonas oryzae pv. oryzae* (*Xoo*), is a worldwide devastating disease, which is second only to the *Pyricularia grisea*, and causes yield losses ranging from 20% to 30%, and in some areas of Asia the loss can be as high as 50% [2].

Traditionally, bacterial blight resistance genes have been cloned by a map-based cloning approach. To date, thirty bacterial blight resistance genes in rice have been identified. Among them, six genes, namely, Xa1, Xa5, Xa13, Xa21, Xa3/Xa26, and Xa27, have been reported to be isolated for bacterial blight resistance [3–6]. While on one hand the results of resistant gene discovery with map-based cloning approach are accurate, these laboratory experiments take years of endeavor and a huge amount of input in terms of human and material resources. It is important to find a more effective way to locate vital resistant genes. 

For a quicker discovery of R genes, the sequence-based approach in bioinformatics is an alternative strategy. In our previous work, Xia et al. [7] presented a novel disease-resistant gene predictor by using chaos games representation (CGR), and the predictor achieved a high accuracy of 98.13% by using a small database with 107 samples. Moreover, Xia et al. also applied this classifier onto the whole KOME database (Knowledge-based Oryza Molecular Biological Encyclopedia, ftp://cdna01.dna.affrc.go.jp/pub/data//20081001/20081001/INE_FULL_SEQUENCE_DB_20081001.zip) and located the top 10 candidate genes, most of which own abundant annotation information in conserved domain information. Unfortunately, direct application of the classifier to the whole database shows a lack of confidence or reliability. 

Additionally, the text mining strategy represents another effective way to improve the efficiency of gene discovery. This strategy usually adopts gene prioritization information among texts to find genes that are possibly related to R gene. For better use of the textual information about the gene, both structural and domain information for *Xoo*-resistant genes should be considered. According to the experimental results in literature [4], most of *Xoo*-resistant genes encode proteins containing conserved nucleotide binding site (NBS) domain and/or leucine-rich repeat (LRR) domain [8] or encode LRR receptor kinase-like proteins. These phenomena suggest a possible internal relation between the gene function and gene structure and offer clues for the text mining strategy [7].

Unfortunately, though both the sequence-based approach and the text mining strategy aim to improve the efficiency of discovery of the targeted R gene against *Xanthomonas oryzae pv. orzyae *(*Xoo*) in rice, the two methods have their own disadvantages. For example, the precision of the sequence-based methods is not high while the recall rate of text mining methods is low. It still has room for enhancement. Henceforth, the purpose of the research to be reported next is to integrate the above two methods into a combined gene discovery strategy so as to achieve a better precision of sequence-based methods and a higher recall of text mining methods as well. 

In this paper, large-scale gene prioritization is enhanced with biomedical text mining technology. After extracting the 31 most distinguished terms in Medline files with term frequency-inverse document frequency, we retrieved 443 candidate proteins with 31 terms. With the classifier built in [7], 74 highly candidate proteins were screened. After searching in Conserved Domains and Protein Classification [9] (http://www.ncbi.nlm.nih.gov/Structure/cdd/cdd.shtml), most of these proteins are proved to be related to *Xoo*-resistant gene in structure and super family information.

## 2. Related Work

Gene discovery based on bioinformatics and text mining are all related to gene prioritization. The definition of a standard definition of gene prioritization is given in [10], that is, given disease D, candidate gene set C, and training data set T; input all these data to a predictor or classifier and the gene prioritization method will compute a score for each of the candidate genes. Genes with higher scores are those with higher probability of being disease D.

According to the type of input data, methods for gene prioritization can be classified into text and data mining methods, as well as network-based methods. Text and data mining methods use training data that includes gene expression [11–13], phenotypic data [14], PubMed abstracts [11], spatial gene expression profiles [12], gene ontology, and other resources [15, 16]. Subsequent computation then will produce scores of candidate genes by mining the genomes or processing currently available biomedical literature. Network-based methods use biological networks [17, 18] as the basis of the prioritization process. There are also network-based methods that combine data and text mining techniques to improve system performance [13, 19]. 

We can also divide current gene prioritization tools into two classes from the perspective of their working principles into functional annotation-based [11, 14, 20–22] and sequence feature based [15, 23]. There are also some studies, like [13], that try to combine these two methods together. Functional annotation tools are usually based on gene expression data. Its underlying principle is that; if a gene is found to be coexpressed with other genes that are involved in a given biological process, this gene can be predicted to be involved in the same process [24]. This principle proceeds from the observation that there is a strong correlation between co-expressions and functional relatedness [24]. The biggest problem for the functional annotation based method is annotation bias, as some genes lack sufficient annotation while others are annotated with abundant information [13]. On the other hand, sequence-based methods utilize information that can be readily computed from the gene sequence, such as gene length, homology and base composition [13]. This method avoids the limitation of annotation bias by making use of intrinsic characteristics of genes. However, it is based on the assumption that these genes have potential involvement in general diseases only rather than some specific disease in which the user is interested [13]. 

Gene seeker [14] is a useful tool to generate a starting list of candidate genes involved in human genetic disorders by gathering positional and expression/phenotypic data from 9 databases automatically. As a controlled vocabulary of anatomical terms, eVOC anatomical system ontology is designed in [11] to integrate clinical and molecular data through a combination of text and data mining methods. The candidate disease genes are selected according to their expression profiles by matching tissues associated with diseases to genes expressed in the tissues. Piro et al. [12] proves that spatially mapped gene expressions are suitable for candidate gene prioritization. The results demonstrate that spatial gene expression patterns have been successfully exploited to predict gene-phenotype associations for both mouse phenotypes and human central nervous system-related Mendelian disorders. 

PROSPECTR [15] is a classifier based on sequence features to rank genes involved in Mendelian and oligogenic disorders. It uses a collection of features representing the structure, content, and phylogenetic extent of candidate genes without prior detailed phenotypic knowledge of the disease. In 2005, SUSPECT [13] combined annotation- and sequence-based approaches to prioritize genes on the principle that genes involved in that disease tend to share the same or similar annotation, so as to reflect common biological pathways. It tries to achieve higher precision of annotation-based methods and the better recall of sequence-based methods through four lines of evidence to score genes, that is, sequence features, extent of coexpressions, domain information, and semantic similarity. 

## 3. Materials and Methods

### 3.1. Data Set Construction

To prepare the data set for literature text mining, texts are collected from NCBI PubMed data base (http://www.ncbi.nlm.nih.gov/pubmed) with MedLine format. In order to evaluate the effectiveness of terms for future extraction, ten sets of Medline texts were collected with different keywords, each of which represented fundamental biological function or event for rice gene/protein in literature. As can be seen from Table 1, the first document has a collection of literature related to binding events for rice, and 1428 hits were found, and the following documents collect corresponding biological event-related papers for rice, including catabolism, expression, localization, phosphorylation, regulation, transcription, all events, *Xoo*-related, and rice-related. Among these features, the first seven represent standard active biomedical events, the eighth one is the sum of the above events, and the last two features focus on *Xoo* gene and rice. In sum, the ten text databases reflect sufficient importance and relevance of the active *Xoo *resistant gene in rice.

### 3.2. Text Mining Based Approach: Choosing Controlled Phrase and Evaluation with Term Frequency-Inverse Document Frequency (TF ∗ IDF)

#### 3.2.1. Preparation of Phrase Dictionary for Candidate Gene Annotation

In order to extract candidate genes from the whole data base, a phrase dictionary for candidate gene annotation is built on the annotation line in FASTA file for rice. A record in its standard format is shown as follows.

 >gi*|*313507159*|*pdb*|*1CCR*|*A Chain A, Structure Of Rice Ferricytochrome C At 2.0 Angstroms Resolution

There are 5 sections of information annotated in each record line.

1. “>gi” indicates the beginning of annotation line in NCBI.
2. “*|*313507159*|*” indicates the accession number in NCBI.
3. “pdb” indicates database Protein Data Bank (http://www.rcsb.org/pdb/home/home.do).
4. “*|*1CCR*|*” indicates the protein name in pdb database.
5. “A Chain A, Structure Of Rice Ferricytochrome C At 2.0 Angstroms Resolution” provides additional description.

In essence, the phrase dictionary collects information that can be automatically extracted from Section 5. The basic principle is to extract meaningful phrases. In the examples above for record 1, the 5th section is “A Chain A, Structure Of Rice Ferricytochrome C At 2.0 Angstroms Resolution”; there are two parts separated by a comma. In these cases, they will be considered as two separate phrases, that is “A Chain A” and “Structure Of Rice Ferricytochrome C At 2.0 Angstroms Resolution”.

However, for those fragments extracted, some are meaningful themselves and some do not have any specific meaning. For example, in record 2, record 3, and record 4, there are “unknown protein”, “hypothetical protein”, and “unnamed protein” used in Section 5 for description. In these cases, they are not collected into the phrase dictionary because they lack specific reference. From the original annotation line of FASTA file for each rice protein, 12037 phrases were chosen on the basis of the above rules.

#### 3.2.2. Phrases Evaluation and Sequences Retrieving

The term frequency-inverse document frequency (TF ∗ IDF) is a statistical measure for evaluating the importance or relevance of a specific word to a document among a series of documents or corpus.

For a given term *t* and a specific document *d* among a series of document *D*, we denote *tf*(*t*, *d*) as term frequency which means the occurrence of term *t* in document *d* and denote *idf*(*t*, *D*) as inverse document frequency; that is,

(1) $$\begin{array}{c} idf\left(t,D\right)=\log\frac{\left|D\right|}{\left|\left\{d\in D:t\in d\right\}\right|+1}. \end{array}$$

Here, *idf*(*t*, *D*) is a measure of the general importance of the term *t* in documents *D*. Meanwhile, the TF ∗ IDF is defined as

(2) $$\begin{array}{c} \text{TF}*\text{IDF}\left(t,d,D\right)=tf\left(t,d\right)\times idf\left(t,D\right). \end{array}$$

The smaller value of TF ∗ IDF shows more relevance between term *t* and document *d*. Therefore, related protein sequences can be retrieved according to vital phrases in conjunction with TF ∗ IDF value, after ranking top vital phrases among phrases in the built dictionary.

### 3.3. Gene Priority with Hybrid Strategy

We combine the text mining strategy and sequence-based approach to propose a hybrid algorithm for gene prioritization. See Algorithm 1.

Here, candidate proteins are chosen according to meaningful annotation screening. Afterwards, the candidate sequences are sent into a built-in classifier, and predictive values will be obtained. This classifier is a sequence-based predictor developed by Jingbo et al. [7] and is available for public use. In this classifier, proteins with a positive value will be regarded as possible *Xoo*-resistant rice gene. 

Those proteins passing both tests in text-mining screening and the built-in classifier are chosen as the highly possible *Xoo*-resistant rice gene. Finally, standard bioinformatics methods are applied onto those positive samples for further evaluation.

Thus, by combining both text mining candidate selection approach and sequence-based classifier, a novel hybrid strategy is proposed for gene priority with a specific function protein.

## 4. Results and Discussion

### 4.1. Experiments Results

As illustrated in Section 3, a phrase dictionary is built based on the annotation file for the whole rice protein sequence. The whole dictionary compromises 12037 terms, and *t*_*i*_ (*i* = 1,2,…, 12037) is the *i*th term, *d*_*j*_ (*j* = 1,2,…, 10) refers to “rice”, “rice event”, “bline”, “catabolism”, “expression”, “localization”, “phosphory”, “regulation”, “transcription”, and “*Xanthomas oryzae versus oryzae*”, respectively, and *D* = *d*_1_, *d*_2_,…, *d*_10_. So TF ∗ IDF (*t*_*i*_, *d*_*j*_, *D*) is counted. The sample results are listed in Table 2. 

In order to screen the key phrases with the most general importance, a voting strategy is used. For each *t*_*i*_ (*i* = 1,2,…, 12037) and *d*_*j*_ (*j* = 1,2,…, 10), TF ∗ IDF (*t*_*i*_, *d*_*j*_, *D*) represents the relevance between *t*_*i*_ and *d*_*j*_, the smaller the value, the higher the relevance, whereas zero means the nonexistence of *t*_*i*_ in *d*_*j*_. For each fixed *d*_*j*_, the value of TF ∗ IDF (*t*_*i*_, *d*_*j*_, *D*) is sorted and the relevance of *t*_*i*_ and *d*_*j*_ is ranked, numbered as Rank (*t*_*i*_, *d*_*j*_, *D*). The voting strategy is to choose *t*_*i*_ which satisfies

(3) $$\begin{array}{c} \#\left\{d_{j,(j=1,2,\ldots ,10)}\;\text{Rank}\left(t_{i},d_{j},D\right)<100\right\}>5, \end{array}$$

where # means the order/scale of the set. By using this voting strategy, only those *t*_*i*_ which are in the top 100 among at least 6 out of 10 documents can be chosen as the key phrases. Taking the construction rule of documents corpus into consideration, the majority agreement of relevance ensures the most general importance of chosen *t*_*i*_.

After voting, thirty key phrases are chosen, which are “CR4”, “thioesterase”, “WRKY2”, “exonuclease-1”, “fibrillarin”, “kinase-like”, “WRKY10”, “WRKY30”, “AML1”, “arginase”, “constans”, “decoy”, “glutaredoxin-like”, “glutathione-S-transferase”, “H2A”, “Metalloendopeptidase”, “PDR20”, “RISBZ5”, “SNF2P”, “YY2”, “CIA”, “CR9”, “EL3”, “MtN21”, “NPKL1”, “prohibitin”, “Ramy1”, “UreD”, “UreF”, and “UreG”, respectively. All of the key phrases with greatest importance are listed in Table 3.

By tracing these key phrases in FASTA annotation, 423 rice proteins are retrieved, each of which includes at least one key phrase in the annotation line. For simplicity and clarity, the result of a small subset with 10 retrieved samples is listed in Table 4. Here, the entries in the first column refer to the NCBI numbers, the second column contains the key phrases, and the third column contains the corresponding gene annotations.

As an example, the GI code for the first sample sequence is 15721862 and its annotation line in FASTA file is “>gi 15721862 dbj BAB68389.1 CR4 [Oryza sativa]”, which includes the phrase “CR4”.

Through the text mining approach, 423 rice protein sequences were chosen as the candidate genes which are regarded as relevant and functionally active. Finally, we test the *Xoo*-resistance for each candidate by using the built-in CGR classifier, and 74 sequences passed the testing procedure. Thus, they show possible positive effects on resistance with the screening ratio of 17.49%. With these 74 proteins, we obtain a candidate gene data set for resistant gene against *Xanthomonas oryzae pv. orzyae* (*Xoo*) in rice. In the following section, we aim to identify its positive resistance so as to obtain useful material for rice breeding.

### 4.2. Validation Evaluation of the Candidate Gene Data Set by Conserved Domain Data and Gene Ontology Matching Results

To evaluate the performance of gene prioritization method, the traditional method is map cloning which is time consuming, as mentioned in Section 1. Therefore, some popular bioinformatics validation methods are used. We use Conserved Domain Data (CDD) [9] and Gene Ontology (GO) [25] to observe information hidden in each gene sequence of candidate gene data set by checking both in conserved construction and function.

First, to observe the structure information of 74 screened proteins, CDD matching results are shown in Table 6. Hits in multidomain and super family in Table 6 clearly show a consistent tendency for the proteins we obtained. Most of the 74 proteins show a high consistency in CDD information. For simplicity and clarity, the domain information of the top 10 proteins is listed as below: the domain hits consist of 6 categories, that is, PLN00113, PKclike super family, LRRNT 2, PLN03150, PKc, and LRRRI, which are closely relevant with leucine-rich repeat or protein kinase. As mentioned in Section 1, most of *Xoo*-resistant genes encode proteins containing conserved nucleotide binding site (NBS) domain and/or leucine-rich repeat (LRR) domain or encode LRR receptor kinase-like proteins. Taking this evidence into account, five domains or super families (PLN03150 excluded) can be regarded as indirect structural evidence for resistance. 

In terms of occurrence of LRR or kinase structure, all of the 10 proteins in Table 5 show consistent evidence, which shows that the genes in candidate gene data set demonstrate a good possibility of being resistant to *Xoo*.

Second, the functional information of the screened proteins is also considered by using the search engine of Gene Ontology (GO) [25], which is a popular bioinformatics ontology aiming at standardizing the representation of gene and gene product attributes across species and databases (http://www.geneontology.org/). GO is also a powerful annotation tool providing a controlled vocabulary of functional terms and describing gene product characteristics. The annotation was performed with BLAST2GO [26, 27] which is based on sequence similarity. For the annotation, the configuration settings are as follows: BLASTP against NCBI nonredundant (nr) protein database, *E*-value filter ≤10^−3^, HSP length cutoff of 33, maximum 20 BLAST hits per sequence to sequence description tool, and annotation cutoff of 55. The sequence distribution results for biological process in GO are listed in Table 6.

As can be seen from Table 6, 50 out of 74 gene sequences are connected with GO terms related to cellular response to stimulus, and the hitting ratio is 67.57%. As cellular response to stimulus is a clear clue for resistant gene, the recall ratio is considerable. Observing entries in the first column of Table 6, which reflect gene function information, there are other five entries relevant to gene resistance, that is, “response to stress”, “response to biotic stimulus”, “response to external stimulus”, “response to abiotic stimulus”, and “response to endogenous stimulus”. Among them, 44 genes are hit for response to stress, 40 for response to biotic stimulus, 37 for response to external stimulus, 33 for response to abiotic stimulus, and 10 for response to endogenous stimulus. These results strongly support the hypothesis that proteins ranked in top list show evidence of resistance response. Since the final validation should be verified by the traditional laboratory experiment, the intensively selected candidate data set holds great potentials worthy of empirical testing and verification.

## 5. Conclusion

**Figure 1:**
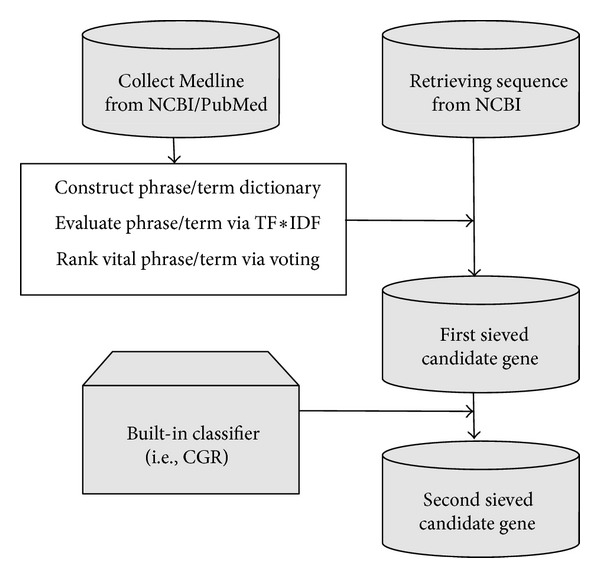
Flowchart of the Hybrid Strategy.

In this research, a hybrid strategy of gene prioritization is proposed, and reasonable results have been obtained. The flowchart of this strategy is shown in Figure 1. The protein sequences and literature texts are both automatically collected from NCBI database, and our scheme consists of two sieves, the text-mining sieve and the classifier sieve. The first sieve is to screen candidate gene according to the important phrase evaluation through TF ∗ IDF and voting scheme. After this step, only those protein sequences with vital annotation are retained in the candidate set. Furthermore, the second sieve is a built-in classifier based on chaos games representation, and sequences predicted to be positive in this step show sufficient sequence similarity with 13 known *Xoo*-resistant proteins. The two sieves represent two popularly used but totally different methods for gene prioritization. After both sieving steps, the remaining sequence corresponds to those highly possible candidate genes. Thus a hybrid strategy for gene prioritization is proposed.

The effectiveness of this hybrid strategy stems from the successful combination of both a sequence-based classifier and text-mining based candidate screening. Generally, for a mere sequence-based predictor, the fraction of retrieved genes relevant to resistance is small, which leads to a low precision value and a high false positive rate. Meanwhile, for a mere text-mining based candidate screening method, the fraction of retrieved genes relevant to resistance is also low, which means a low recall rate. By balancing the high false positive rate and low recall rate, the hybrid strategy proposed in our work achieves a considerably accurate gene screening. The validation test of the candidate dataset shows that our proposed strategy is a significant attempt in large-scale gene prioritization. 

The success of the hybrid strategy also benefits from the abundant information about the targeted gene. On the one hand, the disease resistant gene is quite a popular research model and there has been an increasingly large number of text and sequence resources about R gene. On the other hand, the disease gene resistance possesses many bio-specific properties which make it clear and convenient to locate resistance through texts by using key phrase matching during text mining. 

More significantly, the strategy proposed in this paper is domain free, which means that it shows good potentials for use in other cases for different functional gene prediction. Currently, besides disease resistant gene, like *Xoo* resistant gene, more and more resistant genes are being investigated for better functional annotation or gene discovery, including cold resistant, drought resistant, and herbicide resistant genes. Therefore, the proposed hybrid methods are expected to be highly successful in achieving enhanced gene prioritization.

## Figures and Tables

**Algorithm 1:**
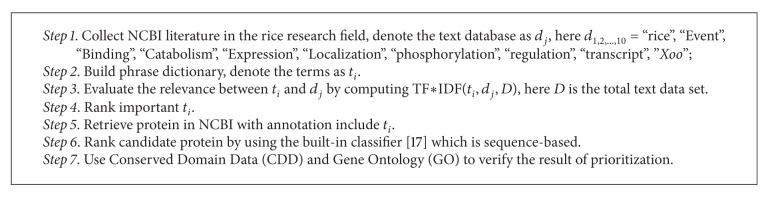
Gene prioritization algorithm.

**Table 1:** Searching strategy for PubMed literature in rice.

Searching content	PubMed hit
Binding	1428
Catabolism	47
Expression	5170
Localization	816
Phosphorylation	226
Regulation	4067
Transcription	2624
All of the above events	6810
*Xanthomonas oryzae pv. oryzae * or *Xoo *	402
(Oryza sativa) or rice	33349

**Table 2:** Sample list of evaluation of vital phrase by TF∗IDF (t_i_, *d*_*j*_, *D*).

*t*_*i*_	*d*_1_	*d*_2_	*d*_3_	*d*_4_	*d*_5_	*d*_6_	*d*_7_	*d*_8_	*d*_9_	*d*_10_
WRKY14	0.79	0.79	0	0	0.79	0	0	0.79	0.79	0
RadA	3.02	2.41	2.41	0	2.41	2.41	0	2.41	0	0
UreD	0.6	0.6	0.6	0.6	0.6	0	0	0.6	0	0
CC-NBS-LRR	4.22	2.41	1.21	0	2.41	0	0	0.6	1.21	0
Urease	18.45	1.35	0.9	0.45	0.9	0	0	0.45	0.45	0
Hd6	7.85	3.02	0	0	3.02	0	0.6	3.02	0.6	0
Carboxypeptidase	15.85	8.56	0.32	0.95	6.02	0.95	0	5.07	0.63	0
EUI	2.2	1.8	0.2	0.2	1.6	0.2	0	1.6	0.6	0.2
H2A	1.9	1.59	0.32	0	0.95	0.32	0.32	0.32	0.95	0
Prolin	34.73	22.11	2.85	0.19	20.97	2.85	0.57	16.99	16.61	0.57
Polypeptide	36.82	18.6	5.69	0.19	14.14	2.85	1.14	8.92	7.78	0.66
Reductase	110.37	47.45	7.21	1.23	37.3	5.31	0.76	26.19	15.75	0.66

(*d*_1,2,…,10_ = “rice”, “event”, “binding”, “catabolism”, “expression”, “localization”, “phosphorylation”, “regulation”, “transcript”, and “*Xoo*”.)

**Table 3:** Voting results of key phrases with greatest importance.

Term	*d*_1_	*d*_2_	*d*_3_	*d*_4_	*d*_5_	*d*_6_	*d*_7_	*d*_8_	*d*_9_	*d*_10_	Vote
CR4	219	7	13	73	7	2	7	3	1	1	9
Thioesterase	106	6	1	63	6	1	6	14	20	8	9
WRKY2	88	62	4	65	74	9	130	91	96	21	9
Exonuclease-1	1	1	14	74	1	20	133	6	6	130	8
Fibrillarin	2	2	15	75	2	21	134	7	7	131	8
Kinase-like	204	149	2	64	76	16	40	16	2	79	8
WRKY10	3	3	16	76	3	247	267	10	9	43	8
WRKY30	4	4	17	77	4	248	268	11	10	44	8
AML1	95	16	42	98	15	254	274	31	29	148	7
Arginase	91	60	19	32	66	292	310	12	11	133	7
Constans	96	17	43	99	16	255	275	32	30	149	7
Decoy	206	5	18	78	5	22	135	8	8	132	7
Glutaredoxin-like	6	9	35	94	11	38	149	20	362	376	7
Glutathione-S-transferase	227	181	32	91	196	17	12	92	3	7	7
H2A	103	145	5	66	75	10	20	4	95	211	7
Metalloendopeptidase	54	15	41	97	14	39	150	21	363	377	7
PDR20	7	10	36	95	12	252	272	29	27	146	7
RISBZ5	40	58	84	138	69	76	175	81	86	203	7
SNF2P	8	11	37	96	13	253	273	30	28	147	7
YY2	41	59	85	139	70	77	176	82	87	204	7
CIA	297	168	33	92	166	4	8	156	22	10	6
CR9	224	61	86	140	71	78	177	83	88	205	6
EL3	71	117	20	79	68	294	47	126	14	136	6
MtN21	55	85	462	463	24	43	153	24	25	144	6
NPKL1	5	8	445	446	22	41	65	17	360	374	6
Prohibitin	202	148	6	67	26	260	92	151	5	20	6
Ramy1	58	88	48	104	99	315	332	37	33	152	6
UreD	9	12	38	38	8	249	269	26	365	379	6
UreF	10	13	39	39	9	250	270	27	366	380	6
UreG	11	14	40	40	10	251	271	28	367	381	6

**Table 4:** The sample of retrieving protein sequences.

NCBI	Term	Annotation
15721862	CR4	>gi 15721862 dbj BAB68389.1 CR4 [Oryza sativa]
56201806	Thioesterase	>gi 56201806 dbj BAD73256.1 putative acyl-(acyl carrier protein) thioesterase [Oryza sativa Japonica Group]
50843956	WRKY2	>gi 50843956 gb AAT84156.1 transcription factor WRKY24 [Oryza sativa Indica Group]
54111120	Exonuclease-1	>gi 54111120 dbj BAD60834.1 exonuclease-1 [Oryza sativa Japonica Group]
18071363	Brillarin	>gi 18071363 gb AAL58222.1 AC09088225 putative brillarin [Oryza sativa Japonica Group]
1586408	Kinase-like	>gi 1586408 prf 2203451 A receptor kinase-like protein
50843970	WRKY10	>gi 50843970 gb AAT84163.1 transcription factor WRKY100 [Oryza sativa Indica Group]
58042751	WRKY30	>gi 58042751 gb AAW63719.1 WRKY30 [Oryza sativa Japonica Group]
52076187	AML1	>gi 52076187 dbj BAD46727.1 putative AML1 [Oryza sativa Japonica Group]
30134457	Arginase	>gi 301344557 gb ADK74000.1 arginase [Oryza sativa Indica Group]

**Table 5:** Multi Domain and Super family Data for Top 10 Sequence in CDD Hit.

Query	Hit type	Short name	Description	Evidence?
Q#1->gi∣53793299	Multidom	PLN00113	LRR	Yes

Q#2->gi∣2586087	Superfamily	PKc_like superfamily	LRR and kinase	Yes
	Superfamily	LRRNT_2 superfamily		
	Multidom	PLN00113		
	Multidom	PLN03150		

Q#3->gi∣343466349	Specific	PKc	LRR and kinase	Yes
	Superfamily	PKc_like superfamily		
	Superfamily	LRRNT_2 superfamily		
	Superfamily	LRR_RI superfamily		
	Multidom	PLN00113		

Q#4->gi∣343466347	Specific	PKc	LRR and kinase	Yes
	Superfamily	PKc_like superfamily		
	Superfamily	LRRNT_2 superfamily		
	Superfamily	LRR_RI superfamily		
	Multidom	PLN00113		

Q#5->gi∣63098460	Superfamily	PKc_like superfamily	LRR and kinase	Yes
	Multidom	PLN00113		

Q#6->gi∣63098462	Superfamily	PKc_like superfamily	LRR and kinase	Yes
	Multidom	PLN00113		

Q#7->gi∣63098474	Superfamily	PKc_like superfamily	LRR and kinase	Yes
	Multidom	PLN00113		

Q#8->gi∣63098472	Superfamily	PKc_like superfamily	LRR and kinase	Yes
	Multidom	PLN00113		

Q#9->gi∣63098486	Superfamily	PKc_like superfamily	LRR and kinase	Yes
	Multidom	PLN00113		

Q#10->gi∣63098454	Superfamily		LRR and kinase	Yes
	Multidom	PLN00113		

**Table 6:** Sequence distribution for biological process in GO database.

Go term	#Seq	Score	Parents	Evidence?
Cellular response to stimulus	50	30	Res, Cep	Yes
Regulation of biological process	50	18	Bir, Bip	
Response to stress	44	44	Res	Yes
Multicellular organismal development	41	72.4	Muo, Dep	
Response to biotic stimulus	40	40	Res	Yes
Primary metabolic process	38	21.4	Mep	
Response to external stimulus	37	37.8	Res	Yes
Anatomical structure development	37	31.2	Dep	
Cell death	34	34	Death, Cep	
Response to abiotic stimulus	33	33	Res	Yes
Establishment of localization	33	19.8	Loc, Bip	
Catabolic process	30	30	Mep	
Reproductive process	30	6.48	Bip, Rep	
Response to endogenous stimulus	10	10	Res	Yes
Macromolecule metabolic process	10	3.6	Mep	
Cellular metabolic process	10	3..42	Mep, Cep	
Cell cycle	5	5	Cep	
Regulation of biological quality	4	0.88	Bir	
Biosynthetic process	3	3	Mep	
Cell communication	3	3	Cep	
Nitrogen compound metabolic process	3	1.08	Mep	
Cellular homeostasis	1	1	Hop, Cep	
